# Sleep dysregulation, memory impairment, and CSF biomarkers during different levels of neurocognitive functioning in Alzheimer’s disease course

**DOI:** 10.1186/s13195-019-0571-3

**Published:** 2020-01-04

**Authors:** Claudio Liguori, Fabio Placidi, Francesca Izzi, Matteo Spanetta, Nicola Biagio Mercuri, Alessandra Di Pucchio

**Affiliations:** 1 Sleep Medicine Centre, Department of Systems Medicine, University of Rome ‘Tor Vergata”, Rome, Italy; 2 Neurology Unit, Department of Systems Medicine, University of Rome ‘Tor Vergata”, Viale Oxford, 81 00133 Rome, Italy; 3 Fondazione Santa Lucia IRCCS, Rome, Italy; 4 Training Office, Italian National Institute of Health (Istituto Superiore di Sanità), Rome, Italy

**Keywords:** Sleep, CSF biomarkers, Alzheimer’s disease

## Abstract

**Background:**

Alzheimer's disease (AD) is frequently accompanied by sleep impairment, which can induce AD-related neurodegeneration. We herein investigated the sleep architecture, cognition, and cerebrospinal fluid (CSF) biomarkers (tau proteins and β-amyloid_42_) during AD progression from subjective cognitive impairment (SCI) to mild cognitive impairment (MCI) and eventually to AD dementia, and compared the results with cognitively normal (CN) subjects.

**Methods:**

We included patients affected by SCI, MCI, mild AD, and moderate-to-severe AD in our study along with CN subjects as controls. All the subjects underwent nocturnal polysomnography to investigate sleep, neuropsychological testing to evaluate cognition, and lumbar puncture for CSF AD biomarkers assessment.

**Results:**

Sleep (both rapid eye movement (REM) and non-REM sleep) and memory function are both progressively impaired during the course of AD from SCI to mild and subsequently to moderate AD. Further, sleep dysregulation appears earlier than cognitive deterioration, with a reduction of CSF β-amyloid_42_ level.

**Conclusion:**

Sleep, memory, and CSF AD biomarkers are closely interrelated in AD progression from the earliest asymptomatic and preclinical stages of the disease related in AD since the earliest and preclinical stages of the disease.

## Introduction

Alzheimer’s disease (AD) is a neurodegenerative disorder characterized by progressive memory loss and a decline in global cognition [1]. A progressive alteration of cognition can be observed in the process of developing AD starting with subjective cognitive impairment (SCI) and progressing to mild cognitive impairment (MCI), which may likely represent the stage preceding AD dementia [2]. Neuropathologically, AD is characterized by progressive accumulation of both extracellular β-amyloid plaques and intracellular neurofibrillary tangles (NFT) of tau proteins, which result in a dramatic loss of neurons and synapses that affect the structural and physiological processes of the brain. Currently, β-amyloid_42_ (Aβ_42_)_,_ total tau (t-tau), and phosphorylated-tau (p-tau) proteins are the established cerebrospinal fluid (CSF) biomarkers that support the diagnosis of AD [3]. The CSF biomarkers of AD, Aβ_42_ in particular, begin to accumulate pathologically in the brain several years before the onset of cognitive deterioration wherein the subjects appear cognitively normal (CN) [4]. In this preclinical stage, however, behavioral modifications such as depression, anxiety, and sleep impairment can appear [5–7]. Accordingly, sleep dysregulation with a reduction of REM and slow-wave sleep may be observed in CN subjects that exhibit biomarkers of AD neurodegeneration [8–10]. Conversely, sleep disorders such as insomnia and obstructive sleep apnea may induce preclinical modifications of AD biomarkers and consequently trigger neurodegeneration by negatively modulating sleep and reducing its beneficial effects on brain health [11–13]. The reduction of sleep quality and an increase in nighttime wakefulness have been hypothesized to cause β-amyloid brain deposits from the early stages of AD [14–16]. Therefore, a bidirectional association seems to exist between sleep dysregulation and AD pathology with both contributing to the progressive alteration of cognitive processes and behavior.

The aim of this study was to investigate nocturnal sleep architecture changes, AD biomarkers in the CSF, and memory impairment across different levels of neurocognitive functioning and impairment ranging from the CN condition to SCI, MCI, and AD dementia.

## Methods

### Patients and study design

We included drug-naïve patients who were consecutively referred to the Neurological Clinic of the University Hospital of Rome “Tor Vergata” between January 2012 and July 2016. Inclusion criterion was patients with a diagnosis of SCI, MCI, or AD according to the recently proposed version of the diagnostic guidelines [1, 2, 17]. All patients underwent a diagnostic and experimental study protocol including history, physical and neurological examination, laboratory tests, standard neuropsychological evaluation, electroencephalogram (EEG), polysomnography (PSG), brain MRI, and lumbar puncture (LP) for CSF analysis. This protocol has been published in previous articles by our research group [11, 18–20].

Specifically, patients were considered affected by SCI if they reported subjective memory deficits reflecting a decline over the past 5 or 10 years, an absence of overt cognitive deficits, and cognitive performance in the normal range [2, 21–23]. The criteria used to define MCI due to AD included the following: cognitive concerns, objective evidence of cognitive impairment, normal functional activities, absence of dementia, and presence of AD biomarkers [17]. The diagnosis of AD was performed according to the recently proposed version of the diagnostic guidelines [1]. The biomarkers were considered as positive for AD when decreased CSF levels of Aβ_42_ were observed along with the following abnormalities: medial temporal lobe atrophy on MRI, cortical temporoparietal hypometabolism on 18F-fluorodeoxyglucose positron emission tomography ([18] FDG PET), and increased CSF levels of t-tau or p-tau [1]. Finally, we divided AD patients into the following two subgroups on the basis of their Mini-Mental State Examination (MMSE) profile: mild AD (mAD, MMSE ≥ 21) and moderate-severe AD (msAD, MMSE < 21) [18].

We enrolled a control group comprising CN subjects who underwent PSG, neuropsychological testing, brain MRI, and LP for diagnostic purposes (to rule out peripheral nerve diseases and/or chronic migraine).

Patients and controls were also required to fulfill the following entry criteria: no additional neurological or psychiatric disease; no intake of CNS active drugs; no use of caffeine, tobacco, and/or alcohol at the time of the sleep laboratory investigation. Contrarily, exclusion criteria for both patients and controls included the following: neoplastic or thyroid illness, diagnosis of conditions interfering with sleep quality such as symptomatic obstructive pulmonary disease, uncontrolled seizures, and abnormal cell count (> 4 cells/mcL) in the CSF sample.

Patients and controls provided their informed consent to the study, which was approved by the Independent Ethical Committee of the University Hospital of Rome “Tor Vergata.” Any anonymized data not published within the article will be shared on request from any qualified investigator.

### Polysomnography

Patients and controls underwent two consecutive video-PSGs in order to evaluate nocturnal sleep (Somnomedics, Somnoscreen, SOMNOmedics GmbH-Randersacker, Germany). The signal was stored on a flash card using a common average reference and a time constant of 0.3 s. Electrodes were positioned according to the 10–20 International System. The montage consisted of two electrooculographic channels, three electromyographic channels (mentalis and anterior tibialis muscles), and eight EEG channels (F4, C4, O2, A2, F3, C3, O1, A1). Cardiorespiratory parameters were assessed by recording oronasal flow, thoracic and abdominal movements (plethysmography), pulse oximetry, and electrocardiogram. Patients and their caregivers were also instructed to maintain the usual sleep schedule and record it in a sleep diary during the week preceding the evaluation. The first-night sleep was considered as an adaptation period. Sleep analysis was performed according to the standard criteria on the second night of PSG monitoring [24]. The following standard parameters were computed: time in bed (TIB; time spent in bed between lights off and lights on), sleep onset latency (SL; the time-interval between the lights off and the first sleep epoch), total sleep time (TST; the actual sleep time without SL and awakenings), sleep efficiency (SE; the ratio between TST and TIB), REM sleep latency (LREM; the time-interval between the sleep onset and the first epoch of REM), stage 1 of non-REM sleep (N1), stage 2 of non-REM sleep (N2), stage 3 of non-REM sleep (N3), REM sleep (REM), and wakefulness after sleep onset (WASO). The percentages of the sleep stages were calculated over TST. Blinded researchers (CL, FP, FI) scored the PSG recordings on the basis of the international standard criteria of the American Academy of Sleep Medicine [24, 25]. PSG scorers also identified apnea/hypopnea events and scored leg movements based on the AASM international standard criteria [24]. Patients with Apnea-Hypopnea Index (AHI) > 15/h and/or periodic leg movement index (PLMI) > 15/h during the polysomnographic recording were ruled out.

### CSF collection and analysis

All the CSF samples were obtained on the day after the second PSG recording by means of LP performed in the decubitus position using an atraumatic needle; the samples were collected between 8:00 AM and 9:30 AM (within 1–2 h after morning awakening) in polypropylene tubes using standard sterile techniques. The first 4 ml CSF sample was used for routine biochemistry analysis including total cell count and lactate levels. The second 4 ml CSF sample was centrifuged to eliminate cells and cellular debris and immediately frozen at − 80 °C to analyze t-tau, p-tau, and Aβ_42_ levels subsequently. CSF biomarkers’ levels were determined according to the previously published standard procedures using commercially available sandwich enzyme-linked immunosorbent assays (ELISA) (Innotest β-Amyloid 1–42, Innotest h-T-tau, Innotest Phospho-T-tau 181, Innogenetics, Ghent, Belgium) [26]. Aβ_42_, t-tau, and p-tau were classified based on previously established cutoff values: < 500 pg/mL for Aβ_42_, > 375 pg/mL for t-tau, and > 52 pg/mL for p-tau [27–29].

### Neuropsychological assessments

The Mini-Mental State Examination (MMSE) was used to screen all participants within 48 h of PSG examination. Administration of the neuropsychological test battery required approximately 20 min, and scores were corrected for age and education level as described in a previous study [30]. Since memory is the most frequently altered domain in AD, we specifically evaluated short- and long-term memory using the Rey Auditory Verbal Learning Test (RAVLT). RAVLT consists of a list of 15 words that are read out to the subject five times. Measures include immediate recall (the sum of the words recalled in the five trials, RAVLT-I) and a 15-min delayed recall (the number of words recalled 15 min after the last word presentation, RAVLT-D).

### Statistical analysis

Demographic and clinical data of patients and controls were reported as frequency (N), percentage (%), mean, standard deviation (SD), and extreme values (minimum and maximum).

A one-way ANOVA was performed to compare sample characteristics according to pathologic or normal cognition profile/diagnosis. Moreover, if the ANOVA was significant, we carried out a post hoc analysis by applying Bonferroni’s correction to identify which groups were different from the others.

Correlation analysis (Pearson correlation coefficients) was conducted to study the strength of the relationship between variables. In particular, we used Cohen’s guidelines (*r* < |.10| negligible association, |.10| < *r* < |.30| weak, |.30| < *r* < |.50| moderate, *r* > |.50| strong) to interpret the effect size of correlations [31]. The significance level (*p* value) was also determined: any *p* value less than 0.05 would indicate that the result is not due to chance.

Data were submitted to principal component analysis (PCA), which was performed on variables chosen as a function of their proven or hypothetical relationship with cognitive profile and degeneration. PCA, a variant of factor analysis, is a data-driven analysis, and the output is a set of “components” with each of them explaining a part of the data variability. The ultimate purpose of PCA is to name and interpret components in physiological or clinical terms. The solution obtained by PCA has a simple format to describe the internal structure of the data set, which explained 62.5% of the total variance in our case [32, 33]. Estimation of the number of components accepted in the PCA solution was based on both Kaiser’s rule, which retains factors with eigenvalues > 1 and Cattell’s scree test, which retrieves the components corresponding to the last eigenvalue before they start to level off. Kaiser–Meyer–Olkin (KMO) Measure of Sampling Adequacy and Bartlett’s Test of Sphericity were used, before the extraction of the component, to assess the suitability of respondent data for PCA. The KMO index ranges from zero to one, with 0.50 considered suitable for factor analysis [34]. Bartlett’s Test of Sphericity should be significant (*p* < 0.05) for factor analysis to be relevant [35]. For the final solution, factors were constrained to remain uncorrelated (i.e., independent), and the solution structure was simplified by using orthogonal axis rotation with the varimax method. Measures were assigned to the component on which they showed the highest loading. We consider loadings > 0.6 to be very high, and loadings < 0.3 to be irrelevant. List-wise deletion was used to handle missing data.

All analyses were performed with Statistical Package for Social Sciences (SPSS) version, 2018. A *p* value less than 0.05 was considered statistically significant.

## Results

### Sample description and demographic data (one-way ANOVA analysis)

Descriptive information (mean, standard deviation, maximal, and minimal values) of the population, which was divided into groups (CN, SCI, MCI, mAD, and msAD) are summarized in Table 1. Altogether, 258 subjects were recruited for this study between January 2012 and July 2016. The whole population was distributed among five groups: two groups of AD patients (56 patients with mild AD and 48 patients with moderate-severe AD), one group of 59 MCI subjects, one group of 54 SCI subjects, and one group of 41 CN subjects.

**Table 1:** Demographic and clinical data of patients and controls. ANOVA was used for the comparisons of variables among the five groups

Demographic and clinical data	Subjects/patients				Controls	Total sample (*n* = 258) (including controls)	F	ANOVA *p*	Post hoc comparisons
	(2) SCI (*n* = 54)	(3) MCI (*n* = 59)	(4) m-AD (*n* = 56)	(5) ms-AD (*n* = 48)	(1) CN (*n* = 41)				
Gender									
Male, *N* (%)	24 (44.4)	28 (47.4)	21 (37.5)	19 (39.6)	20 (48)	112 (43)			
Female, *N* (%)	30 (55.6)	31 (52.6)	35 (62.5)	29 (60.4)	21 (52)	146 (57)			
Age, mean ± SD (range)	64.94 ± 8.75 (44–80)	67.42 ± 8.43 (44–80)	69.93 ± 7.27 (55–83)	71.71 ± 7.19 (53–82)	67.17 ± 9.83 (44–83)	68.21 ± 8.55 (44–83)	5.13	.001	2 < 4,5
MMSE score, mean ± SD	29.11 ± 1.00 (27–30)	25.85 ± 1.51 (23–29)	24.45 ± 1.85 (21–27)	15.40 ± 3.21 (4–20)	29.20 ± 0.90 (27–30)	24.81 ± 5.22 (4–30)	433.98	.000	5 < 4 < 3 < 2,1
CSF biomarkers									
T-tau (pg/mL)	253.67 ± 163.87 (92–1117)	569.59 ± 363.82 (160–1876)	632.07 ± 243.18 (116–1190)	744.44 ± 254.34 (222–1245)	234.22 ± 64.42 (116–402)	496.26 ± 317.21 (92–1876)	41.87	.000	1,2 < 3 < 5; 1,2 < 3,4
P-tau (pg/mL)	42.37 ± 23.60 (4–156)	85.41 ± 49.04 (32–240)	88.75 ± 35.15 (31–189)	100.77 ± 45.42 (34–235)	42.34 ± 11.76 (22–85)	73.14 ± 43.60 (4–240)	27.80	.000	1,2 < 3,4,5
Aβ_42_ (pg/mL)	666.44 ± 257.75 (259–1123)	320.22 ± 98.76 (132–460)	275.84 ± 68.42 (82–440)	270.67 ± 90.95 (123–463)	854.66 ± 182.15 (582–1197)	458.76 ± 275.60 (82–1197)	140.48	.000	5,4,3 < 2 < 1
Neuropsychological tests									
RAVLT-I score, mean ± SD	44.31 ± 3.91 (39–54)	23.27 ± 2.95 (18–28)	18.30 ± 3.36 (11–23)	11.54 ± 2.98 (7–16)	46.20 ± 4.44 (39–53)	28.06 ± 14.04 (7–54)	956.40	.000	5 < 4 < 3 < 2,1
RAVLT-D score, mean ± SD	9.87 ± 2.01 (7–14)	2.80 ± 1.23 (0–4)	1.57 ± 1.17 (0–4)	1.25 ± 1.02 (0–3)	10.46 ± 1.58 (8–13)	4.94 ± 4.25 (0–14)	495.32	.000	5,4 < 3 < 2,1
Polysomnography									
TST (min)	348.070 ± 79.86 (161.50–580)	349.37 ± 53.65 (146.5–415)	318.82 ± 44.16 (220–430)	252.93 ± 47.77 (149.3–361.8)	367.22 ± 60.93 (135–444)	327.36 ± 69.88 (135–580)	28.19	.000	5 < 4 < 2,3,1
SE (%)	77.80 ± 13.53 (31.5–96.4)	78.50 ± 10.55 (50.4–94)	74.4 ± 6.71 (59.4–89)	67.76 ± 9.21 (36.5–91.5)	89.15 ± 2.27 (84.3–92.4)	77.16 ± 11.42 (31.5–96.4)	29.64	.000	5 < 4,3 < 1; 5 < 2 < 1
SL (min)	23.30 ± 64.62 (0.30–343)	27.73 ± 36.02 (2–150)	20.46 ± 16.63 (1–76)	24.27 ± 23.70 (1–122)	12.95 ± 6.34 (5–36)	22.23 ± 36.67 (0.30–343)	1.07	.372	NS
REML (min)	119.49 ± 78.97 (40–412)	135.25 ± 82.13 (12–330)	179.64 ± 102.92 (12–424)	143.57 ± 51.32 (27.5–220)	103.83 ± 16.16 (77–143)	138.14 ± 79.00 (12–424)	7.27	.000	1 < 3; 1,2 < 4
N1 (%)	16.07 ± 7.00 (3.8–34)	21.87 ± 8.18 (7.5–34.6)	31.05 ± 8.61 (12.2–45.7)	33.81 ± 11.00 (10.4–64.1)	15.37 ± 3.35 (10–21)	23.84 ± 10.95 (3.80–64.1)	53.50	.000	1,2 < 3 < 4,5
N2 (%)	55.10 ± 7.66 (34–72.6)	52.12 ± 8.18 (31.8–67)	51.35 ± 9.41 (31.8–71.7)	54.67 ± 9.02 (28.10–76)	50.90 ± 3.45 (44–59)	52.86 ± 8.11 (28.10–76)	2.92	.022	3 < 2
N3 (%)	16.29 ± 6.64 (6.5–29.3)	16.14 ± 5.85 (4–31.4)	11.18 ± 7.70 (0.80–28.7)	6.93 ± 5.18 (0–22)	17.83 ± 1.46 (14–21)	13.65 ± 7.08 (0–31.4)	28.11	.000	5 < 4 < 3,2,1
REM (%)	12.56 ± 5.62 (0–25.5)	9.37 ± 4.99 (0–20)	6.41 ± 3.94 (0.80–16)	4.60 ± 3.69 (0–12.20)	15.90 ± 2.96 (11–22)	9.54 ± 5.86 (0–25.5)	49.41	.000	5 < 4 < 3 < 2 < 1
WASO (min)	99.48 ± 67.54 (17.5–351.2)	78.54 ± 56.61 (− 69.19–221)	92.26 ± 42.82 (− 8.52–193.45)	97.63 ± 47.88 (4.74–240.72)	31.95 ± 12.42 (3.74–53.23)	82.05 ± 55.09 (− 69.19–351.20)	13.54	.000	1 < 3,4,5,2
TIB	451.31 ± 103.92 (274–964)	446.22 ± 51.35 (290–7-600)	431.55 ± 66.51 (269–573.33)	374.83 ± 58.69 (239–498)	412.13 ± 67.27 (146.7–498.9)	425.40 ± 76.53 (146.7–964.15)	9.35	.000	5 < 2; 5 < 3; 5 < 4

*N* frequency, *%* percentage, *Min-Max* extreme value, *SD* standard deviation. The numbers in parentheses in group names refer to the numbers used in illustrating statistically significant differences

*Abbreviations*: *MMSE* Mini-Mental State Examination, *T-tau* total tau, *P-tau* phosphorylated tau, *Aβ*_*42*_ β-amyloid_42_, *RAVLT-I* Immediate Recall – Rey Auditory Verbal Learning Test, *RAVLT-D* Differite Recall – Rey Auditory Verbal Learning Test, *TST* total sleep time, *SE* sleep efficiency, *SL* sleep onset latency, *REML* REM sleep latency, *N1* stage 1 of non-REM sleep, *N2* stage 2 of non-REM sleep, *N3* stage 3 of non-REM sleep, *REM* REM sleep, *WASO* wakefulness after sleep onset, *TIB* time in bed, *NS* not significant, *AD* Alzheimer’s disease, *mAD* mild Alzheimer’s disease, *msAD* moderate-severe Alzheimer’s disease, *MCI* mild cognitive impairment, *SCI* subjective cognitive impairment, *CN* cognitively normal

### One-way ANOVA analysis

Results from one-way ANOVA, which was performed to analyze and compare the included variables from the five groups of subjects, are presented in Table 1. With the exception of the SL variable (*F* = 1.07, *p* = 0.372), there were statistically significant differences between the groups, as determined by one-way ANOVA, for all the included variables.

To confirm whether differences occurred between groups, a post hoc analysis with Bonferroni’s correction was conducted, and the results are shown in Table 1.

#### CSF data (one-way ANOVA analysis)

**Fig. 1:**
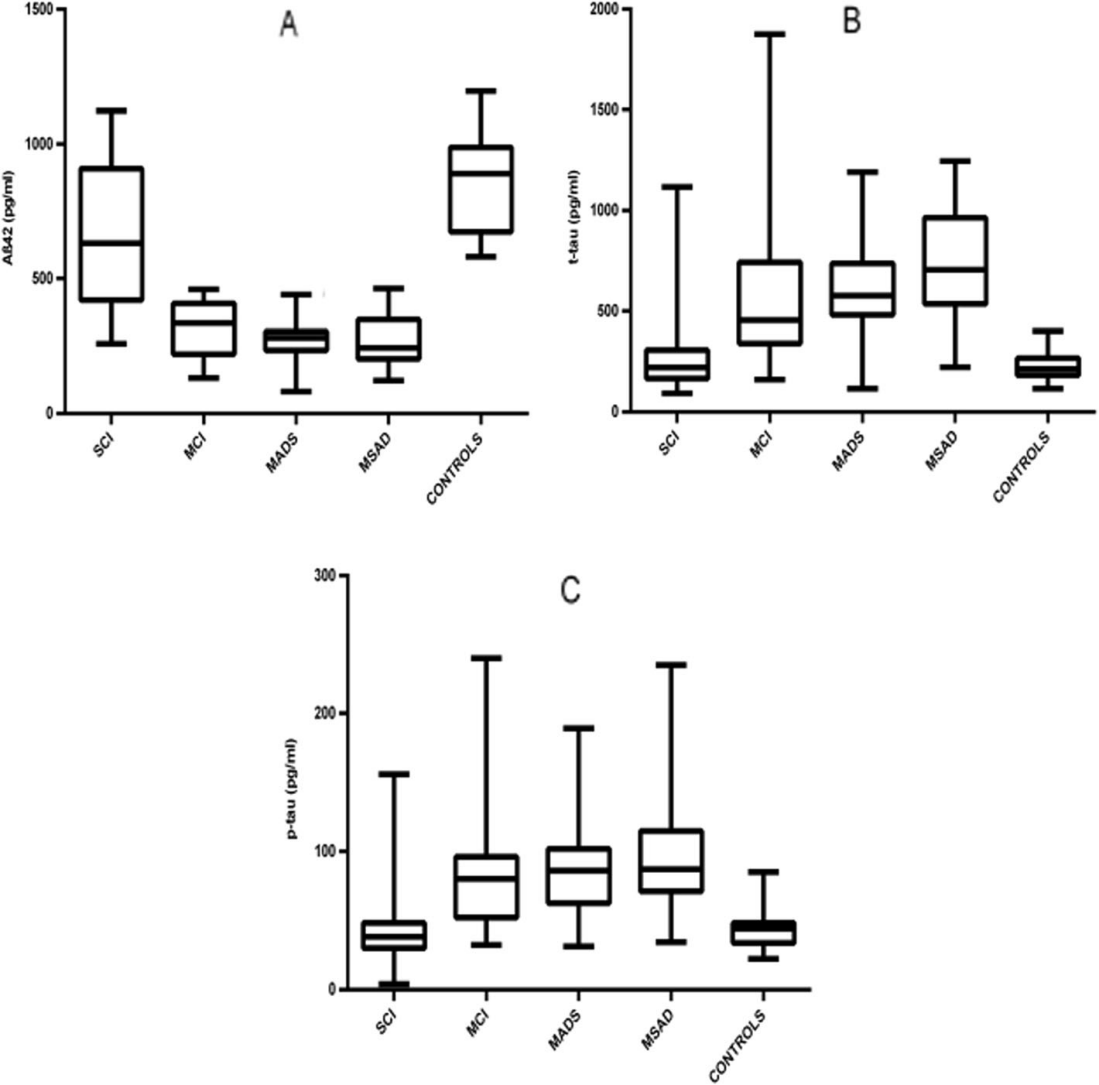
Graphical representation of ANOVA among SCI, MCI, mAD, msAD, and control groups. Box and whisker plots show the median (lines), 25th to 75th percentiles (boxes), and full spread (whiskers) of all the variables. **a** CSF Aβ_42_ levels. **b** CSF t-tau levels. **c** p-tau levels

With reference to CSF biomarkers, we documented a significant reduction of CSF Aβ_42_ levels in MCI, mAD, and msAD patients compared to SCI and CN subjects (Fig. 1). Moreover, SCI patients presented significantly lower CSF Aβ_42_ levels than the CN subjects did (Table 1). CSF t-tau and p-tau levels did not differ between SCI and CN, but it was lower in these two groups than in MCI, mAD, and msAD groups (Table 1, Fig. 1).

#### PSG data (one-way ANOVA analysis)

**Fig. 2:**
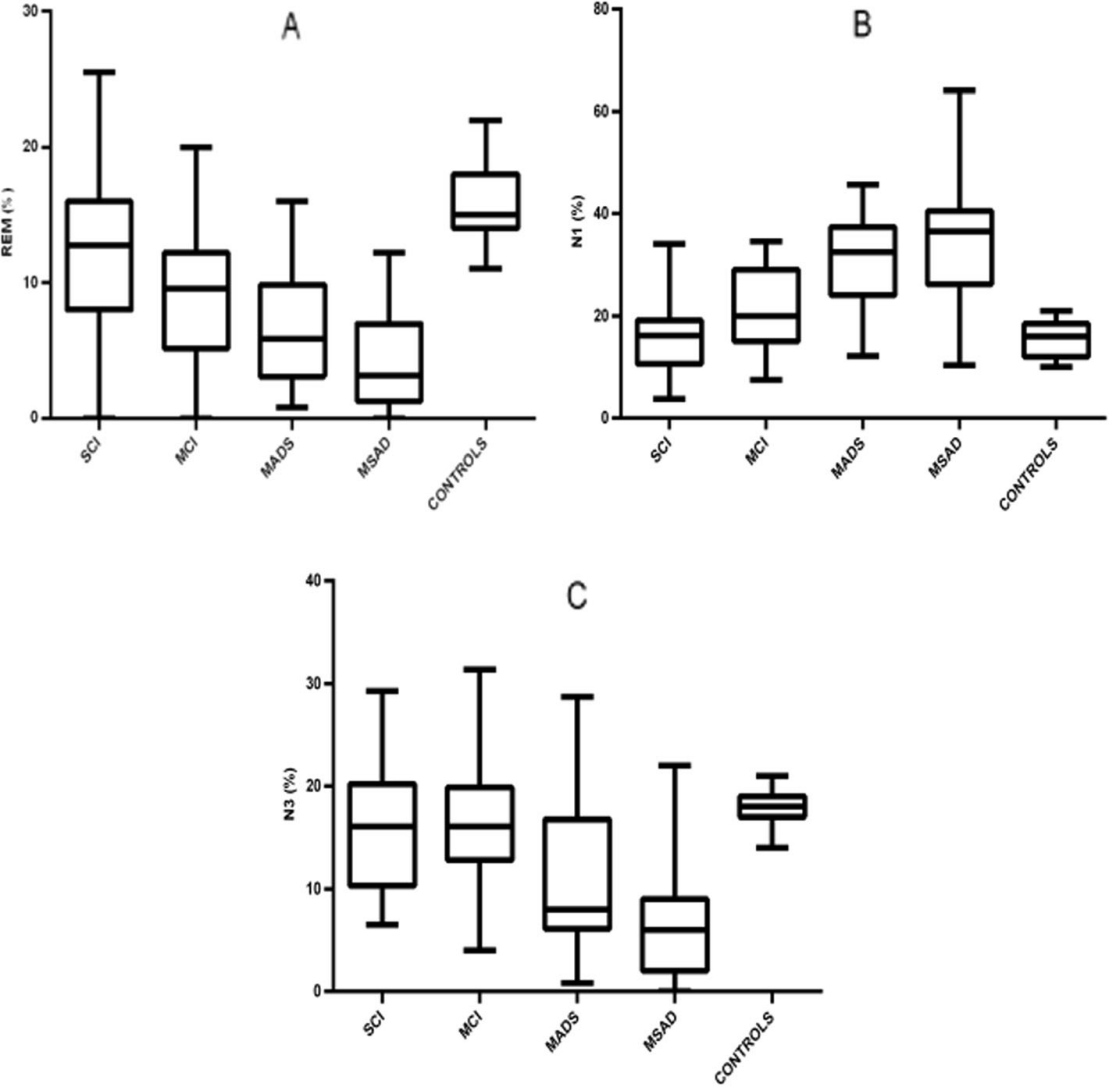
Graphical representation of ANOVA among SCI, MCI, mAD, msAD, and control groups. Box and whisker plots show the median (lines), 25th to 75th percentiles (boxes), and full spread (whiskers) of all the variables. **a** REM sleep. **b** Stage 1 of non-REM sleep (N1). **c** Stage 3 of non-REM sleep (N3)

Analysis of all the PSG variables included in this study was also performed (Table 1). Regarding TIB, we documented a significantly higher TIB in mAD, SCI, and MCI patients compared to msAD. Considering each PSG variable, we observed a significant reduction of both REM sleep and SE in SCI patients compared to CN subjects without any other significant difference in the remaining sleep macrostructural parameters (Fig. 2). TST was lower in CN, SCI, and MCI compared to mAD patients who in turn showed a lower TST than msAD patients. SE was higher in CN subjects than in SCI patients and then became progressively lower in MCI, mAD, and msAD patients. The mAD patients showed the highest LREM compared to SCI and CN subjects. N1 was higher in mAD and msAD patients than in MCI patients who in turn showed a higher N1 compared to SCI and CN subjects (Fig. 2). N3 was lower in msAD compared to mAD patients who themselves showed lower N3 than the MCI and SCI patients, and CN subjects (Fig. 2). REM sleep was significantly reduced in both mAD and msAD compared to MCI patients; moreover, MCI patients showed a significantly lower REM sleep compared to SCI patients who themselves showed reduced REM sleep compared to CN subjects. Finally, WASO was higher in all the pathological groups (SCI, MCI, mAD, and msAD) compared to the CN group.

#### Neuropsychological data (one-way ANOVA analysis)

As expected, a significant reduction of MMSE scores was observed in MCI, mAD, and msAD patients compared to both SCI and CN subjects; moreover, MCI patients presented significantly higher MMSE scores than mAD and msAD patients, and the mAD group presented significantly higher MMSE scores compared to the msAD group (Table 1).

**Fig. 3:**
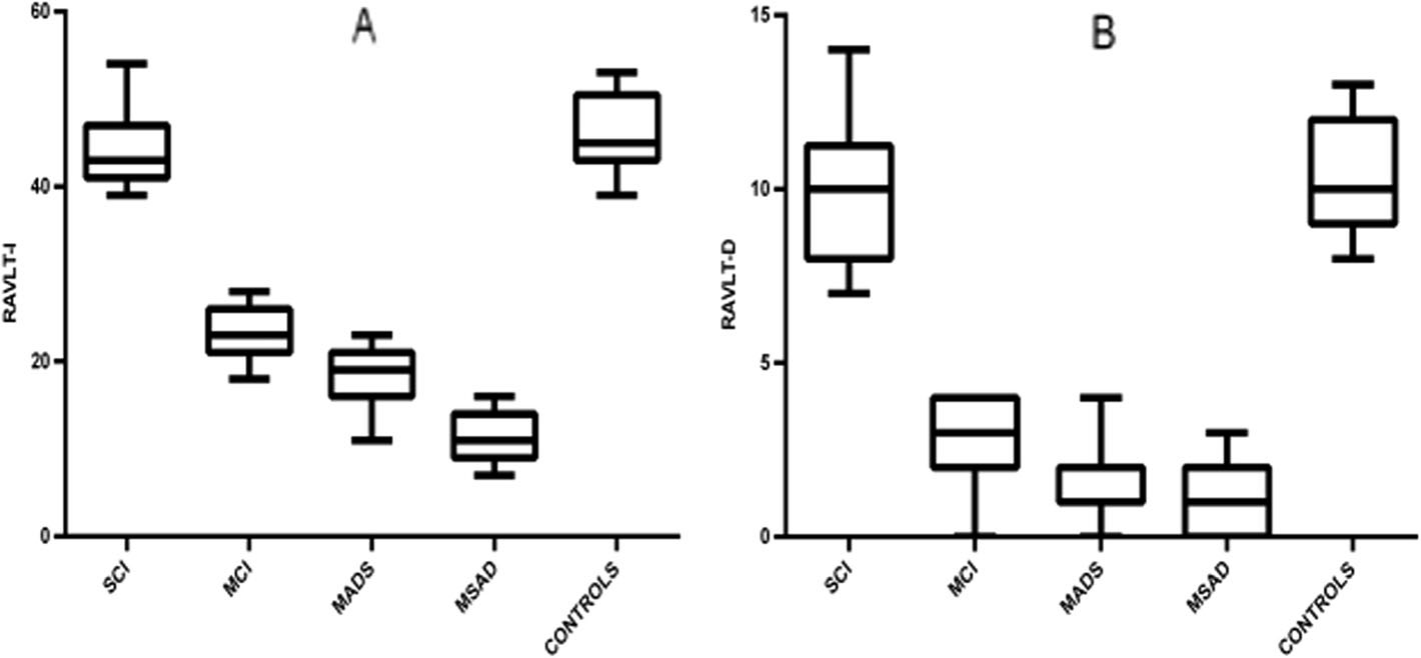
Graphical representation of ANOVA among SCI, MCI, mAD, msAD, and control groups. Box and whisker plots show the median (lines), 25th to 75th percentiles (boxes), and full spread (whiskers) of all the variables. **a** Rey Auditory Verbal Learning Test (RAVLT) – Immediate Recall (RAVLT-I). **b** RAVLT – Delayed Recall (RAVLT-D)

Concerning the tests investigating memory, a significant progressive reduction of RAVLT-I scores was observed in MCI, mAD, and msAD patients compared to SCI and CN subjects (Fig. 3). Considering RAVLT-D scores, the comparison between mAD and msAD groups did not show significant differences, but RAVLT-D scores of mAD and msAD patients were lower than MCI patients, who in turn showed lower scores than SCI and CN subjects (Fig. 3). Notably, SCI patients did not show pathological scores in RAVLT-D and RAVLT-I tests (Table 1).

### Correlation analysis

The correlation matrix shown in Table 2 contains the Pearson correlation coefficients (and the significance level) between the variables, which denotes the strength of the relationship between the variables. Overall, moderate to strong correlations (*r* ≥ 0.50) were found between CSF biomarkers and both neuropsychological and polysomnography data (Table 2).

**Table 2:** Correlation matrix among 15 variables in the whole population (*n* = 258)

Pearson’s correlation	1	2	3	4	5	6	7	8	9	10	11	12	13	14	15	16	17	18
1. Sex	1																	
2. Age	−0.051	1																
3. MMSE	− 0.060	− .209^**^	1															
4. T-tau	0.036	.330^**^	− .488^**^	1														
5. P-tau	−0.019	.299^**^	− .408^**^	.845^**^	1													
6. Aβ_42_	0.010	− .229^**^	.543^**^	− .515^**^	− .422^**^	1												
7. TST	−0.085	− .141^*^	.531^**^	− .324^**^	− .287^**^	.306^**^	1											
8. SE	−0.062	− .224^**^	.471^**^	− .361^**^	− .329^**^	.403^**^	.589^**^	1										
9. SL	0.078	0.066	−0.035	0.103	0.112	− 0.029	0.078	− .292^**^	1									
10. LREM	0.020	0.052	− .161^**^	0.079	0.082	− .260^**^	− .176^**^	− .289^**^	.145^*^	1								
11. N1	0.037	.178^**^	− .538^**^	.434^**^	.351^**^	− .564^**^	− .397^**^	− .428^**^	0. .075	.247^**^	1							
12. N2	0.059	−0.041	−0.070	−0.077	−0.085	0.006	−0.048	0.003	−0.077	.209^**^	− .326^**^	1						
13. N3	−0.095	−0.083	.463^**^	− .333^**^	− .267^**^	.362^**^	.323^**^	.215^**^	0.047	− .185^**^	− .618^**^	− .404^**^	1					
14. REM	−0.025	− .197^**^	.537^**^	− .315^**^	− .256^**^	.613^**^	.420^**^	.555^**^	−0.096	− .513^**^	− .658^**^	− .263^**^	.491^**^	1				
15. WASO	0.041	.151^*^	− .202^**^	.142^*^	.128^*^	− .264^**^	− .268^**^	− .847^**^	.134^*^	.230^**^	.251^**^	−0.008	−0.063	− .405^**^	1			
16. TIB	−0.034	0.000	.272^**^	−0.115	−0.095	0.033	.698^**^	− .146^*^	.386^**^	0.058	−0.116	−0.077	.226^**^	0.039	.428^**^	1		
17.RAVLT-I	−0.037	− .236^**^	.765^**^	− .628^**^	− .546^**^	.802^**^	.429^**^	.457^**^	−0.075	− .270^**^	− .647^**^	0.013	.454^**^	.650^**^	− .209^**^	.156^*^	1	
18. RAVLT-D	−0.045	− .233^**^	.658^**^	− .597^**^	− .505^**^	.828^**^	.353^**^	.444^**^	−0.074	− .264^**^	− .589^**^	0.008	.388^**^	.630^**^	− .238^**^	0.074	.928^**^	1

*p < 0.05; **p < 0.01

*Abbreviations*: *MMSE* Mini-Mental State Examination, *T-tau* total tau, *P-tau* phosphorylated tau, *Aβ*_*42*_ β-amyloid42, *RAVLT-I* Immediate Recall – Rey Auditory Verbal Learning Test, *RAVLT-D* Differite Recall – Rey Auditory Verbal Learning Test, *TST* total sleep time, *SE* sleep efficiency, *LREM* REM sleep latency, *N1* stage 1 of non-REM sleep, *N2* stage 2 of non-REM sleep, *N3* stage 3 of non-REM sleep, *REM* REM sleep, *WASO* wakefulness after sleep onset, *TIB* time in bed, *SL* sleep onset latency

### Principal component analysis

PCA was performed on the following: age, scores of MMSE, RAVLT-I, RAVLT-D, CSF Aβ_42_ level, t-tau and p-tau levels, and a standard set of polysomnography parameters including SL, TST, LREM, N1, N3, REM, and WASO. Some variables were not considered including gender, which was not significantly associated with any variable, N2 because it presented split loadings on all factors, and SE and TIB as they were computed from other polysomnographic parameters. Was excluded as it presented split loadings on all factors, and SE and TIB were excluded as they were computed from other polysomnographic parameters. A three-component solution that explained 62.5% of the total variance was identified by performing PCA with varimax rotation. The KMO measure of sampling adequacy met the “meritorious” criterion (KMO = 0.86) to perform the principal component analysis. Moreover, for these data, Bartlett’s test of Sphericity was highly significant (*p* < 0.001).

The contribution of the three factors to sample variance was 53.5%, 18.1%, and 11.9%.

Factor solution after varimax rotation is presented in Table 3. Overall, with the exception of a few cross-loading variables, each factor defines a distinct cluster of interrelated variables.

**Table 3:** Principal component analysis: factorial solution after varimax rotation

	Factor loadings		
	Component 1	Component 2	Component 3
RAVLT-I	*0.777*	−0.477	
N1	*−0.762*		
MMSE	*0.737*	−0.320	
REM	*0.734*		−0.515
N3	*0.729*		
RAVLT-D	*0.715*	−0.475	
Aβ_42,_ pg/ml	*0.675*	−0.393	
TST	*0.600*		
P-tau pg/ml		*0.823*	
T-tau pg/ml	−0.393	*0.819*	
Age		*0.548*	
REML			*0.664*
WASO			*0.641*
SL		0.328	*0.599*

Note. 1 The table represents the loads of each variable in each of the three factors extracted by PCA. Loads < 0.30 are not presented. Factor loadings over .40 appear in italics

*Abbreviations*: *MMSE* Mini-Mental State Examination, *T-tau* total tau, *P-tau* phosphorylated tau, *Aβ*_*42*_ β-amyloid42, *RAVLT-I* Immediate Recall – Rey Auditory Verbal Learning Test, *RAVLT-D* Differite Recall – Rey Auditory Verbal Learning Test, *TST* total sleep time, *LREM* REM sleep latency, *N1* stage 1 of non-REM sleep, *N3* stage 3 of non-REM sleep, *REM* REM sleep, *WASO* wakefulness after sleep onset, *SL* sleep onset latency

Contribution to *component 1* mainly derived from RAVLT-I (0.78), N1 (− 0.76), MMSE (0.74), REM (0.73), N3 (0.73), RAVLT-D (0.71), Aβ_42_ (0.67), and TST (0.60). Notably, N1 load on the first component was of the opposite sign in comparison to other variables thereby suggesting an inverse correlation among them. *Component 2* explains the association between CSF p-tau (0.82), t-tau (0.82), and age (0.55). Contribution to *component 3* mainly derived from REML (0.66), WASO (0.64), and SL (0.59).

## Discussion

Different lines of evidence suggested that AD neurodegeneration starts several years before the appearance of clinical symptoms, which are not exclusively cognitive but also behavioral (depression, anxiety, sleep fragmentation) [36]. Conversely, insomnia and sleep-disordered breathing (SDB) that frequently affect the elderly have recently been associated with an increased risk of developing AD. This association between sleep disorders and AD has been supposed based on the evidence that sleep dysregulation can induce pathological changes in β-amyloid and tau metabolism in the brain [11–16]. Moreover, in healthy subjects, it has been demonstrated that not just chronic but even a single night of sleep deprivation can alter CSF levels of Aβ_42_ in addition to reducing cognitive performances [37, 38]. Therefore, it is currently under debate if sleep alteration represents a symptom or a possible cause of AD.

In this comprehensive study, we demonstrated that sleep impairment and dysregulation is present before the clinical appearance of objective cognitive deterioration and dementia by analyzing PSG recordings, assessing CSF biomarkers, and performing cognitive tests in subjects ranging from CN to AD dementia. In particular, subjects complaining of SCI already showed pathological modification of sleep architecture (SE, REM, and WASO), which was significantly different from that of CN controls. Moreover, we documented that REM sleep is altered in the preclinical stage of AD and that it is linked to β-amyloid pathology and memory loss.

To better interpret the findings of this study, we applied an exploratory PCA to analyze simultaneously the relationship between sleep and the variables putatively related to cognitive profile and neurodegeneration. The three components obtained by PCA were relatively “pure” with respect to the variables that loaded in each of them, and each variable tended to load heavily in only one component thus making the attribution of the physiological value to the factorial solution relatively easy.

An understanding of the temporal sequence between alterations in sleep architecture and dementia onset remains inadequate in the scientific literature. Accordingly, sleep impairment has been considered as either an early marker of AD pathology or a risk factor for AD. Following the first hypothesis, cross-sectional studies demonstrate that sleep architecture not only progressively deteriorates in patients with dementia but can also be dysregulated from the earlier stages of cognitive impairment [18, 39]. In accordance with the second supposition, REM sleep dysregulation with an increase in latency and a reduction in quantity has recently been associated with the incipient risk of dementia in CN subjects [10]. Further, sleep disturbances such as SDB and insomnia have been associated with the incipient risk of cognitive impairment and AD [8, 11, 14]. Following this line of evidence, orexinergic system dysregulation, sleep-wake cycle impairment, behavioral disturbances, and impaired generation of slow-wave sleep oscillations have been associated with cortical β-amyloid pathology and AD [12, 13, 18–20, 40–42]. Moreover, sleep influences the generation and clearance of β-amyloid by the aggregation of isoform 42 into oligomers and the deposition of brain plaques [43–45]. All these effects seem to be mediated by the functioning of the glymphatic system, which ensures the clearance of extracellular beta-amyloid and other toxic substrates during sleep [12].

Progressive cognitive deterioration and memory loss are the main clinical features of AD pathology. However, several studies suggest that sleep impairment is also a frequent and highly disruptive neuropsychiatric symptom associated with AD [6]. Epidemiological studies have documented that sleep disturbances occur in several patients affected by AD, and increase in frequency with the progression of the disease [6, 46]. Additionally, sleep disturbances can precipitate dementia symptoms with a negative impact on the cognitive and behavioral domains [6, 18]. After a thorough literature purview on the mutual interference of sleep, CSF AD biomarkers, and memory functioning, we examined not only whether sleep architecture is associated with the progression of AD in subjects affected by different stages of AD pathology, but also whether the modifications of sleep architecture correlated with cognitive performances and CSF AD biomarkers.

For this purpose, we performed PCA and documented the mutual interplay among a combination of variables related to sleep architecture (REM, N1, N3, and TST), CSF Aβ_42_ levels, and both global cognitive functioning and memory impairment (MMSE, RAVLT-I, and RAVLT-D) in *Component 1*. Our finding suggests a strong association between these parameters, but it does not give any indication on the possible causal link among them. Therefore, *component 1* linking sleep to CSF β-amyloid concentrations and cognition during the progressive stages of the AD process along with the correlations supplemented the previously hypothesized association between AD pathological biomarkers and sleep dysregulation. Furthermore, it confirms the evidence that sleep fragmentation with reduced REM and N3 sleep is associated not only with β-amyloid pathology but also with tau neurodegeneration [10, 42]. Beyond the already stated association between sleep and cognition, the present findings additionally link β-amyloid and tau neurodegeneration to sleep dysregulation and cognitive impairment in the AD process. Notably, brain regions and networks involved in the control of the sleep-wake rhythm can be affected by AD pathology, which is often clinically complicated by both circadian rhythm disruption and sleep disturbances including night-time awakenings and non-REM/REM sleep dysregulation [3, 47, 48]. *Component 2* of the PCA reflected a combination of variables related to CSF levels of the biomarkers attributed to both neurodegeneration (t-tau and p-tau) and the patient’s age. Age represents a risk factor for AD and cognitive deterioration [43]. Low Aβ_42_ and high t-tau and p-tau concentrations in the CSF are biomarkers of AD, reflecting brain deposition of amyloid plaques and NFT. Since the concentration of CSF biomarkers of AD is associated with age, following the second component of PCA, we confirmed prior evidence that suggested that the association between aging and tau pathology also influences memory performance [43].

Finally, *component 3* of the PCA combined three sleep measures (REML, WASO, and SL) related to sleep quality/fragmentation; this finding further supports our hypothetical model on the role of sleep fragmentation and night-time wakefulness, which can reduce the beneficial effects of sleep against the neurodegenerative processes. Consistently, sleep fragmentation and related nocturnal arousals are accompanied by a significant increase in N1 that is associated with a decrease in N3 and REM. Further, more detrimental effects on sleep are induced by sleep fragmentation than partial sleep deprivation thus supporting the evidence that the brain tolerates sleep deprivation better than sleep fragmentation [44, 49]. Accordingly, sleep fragmentation is associated with cognitive decline and the risk of subsequent AD [50].

Our results consistently demonstrate that REM sleep dysregulation is associated with a more marked damage in the sleep architecture (featured by a more consistent sleep fragmentation) that worsens during the progression of AD. These findings coupled with those of the previous investigation that showed that REM sleep deterioration is associated with the increase in dementia risk further suggests that future research should take into account the role of REM sleep in the pathology of AD [10]. Nevertheless, the mechanisms linking REM sleep to the AD process remain to be well understood. The loss of cholinergic function may underpin REM sleep impairment during the AD process since cholinergic neurons are important determinants of REM sleep [51]. Loss of cholinergic function, degeneration of cholinergic projections in the basal forebrain, and changes in acetylcholine release are established fingerprints of AD neurodegeneration [52]. Moreover, the orexinergic system is found to be dysregulated in AD in both animal models and human studies [18, 53]. According to this evidence, orexinergic signaling malfunction can be related to REM sleep dysregulation thus promoting sleep architecture damage and β-amyloid pathology [18, 53]. Therefore, damage in the cholinergic pathway may cause a dysregulation of the orexinergic system, and both systems may negatively influence sleep, cognition, and AD neurodegenerative processes.

The novelties of this study include the large sample size of patients experiencing different stages of AD and the comparison to a control group comprising CN subjects. All the subjects underwent a very comprehensive protocol evaluating sleep, global cognition, memory functioning, and CSF AD biomarkers. Our study was the first to include subjects affected by SCI, which can represent a very early preclinical stage of AD pathology in humans and to compare them not only to MCI and AD patients but also to CN subjects. Pertinently, SCI was recently defined as a clinical condition indicating an increased risk of AD progression in patients especially when biomarkers consistent with AD are present [2]. Notably, our study documented both non-REM and REM sleep dysregulation during the AD process from the earliest stages of the disease onwards. Finally, the PCA gives further clarity to this data since it allows us to globally analyze all the parameters and interpret them as reciprocally related. In particular, sleep, cognition, and CSF AD biomarkers’ levels appear to be mutually related throughout the different stages of AD; this indicates that sleep is a potential therapeutic target for disease-modifying strategies. Conversely, the main limitation of this study is the non-longitudinal evaluation of the sample subjects.

## Conclusion

Through exploring the interplay between different parameters in our model including not only patients affected by AD at different stages but also CN subjects, we identified three different and independent *components* manifesting a strong relationship among all the analyzed parameters. These include the following: *component 1* linking sleep architecture, neurocognitive and memory functioning, and Aβ_42_ and tau proteins levels, *component 2* linking aging to neurodegeneration, and *component 3* linking sleep fragmentation to REM sleep dysregulation. Hence, this study suggests that sleep dysregulation is not just a risk factor but may also serve as an early marker of AD. To confirm this evidence, a long-term, longitudinal study on patients affected by SCI is necessary in order to track and understand the modifications of sleep, cognition, and neurodegenerative biomarkers.

## Data Availability

Any anonymized data not published within the article will be shared by request from any qualified investigator.
