# Correction to: Sleep dysregulation, memory impairment, and CSF biomarkers during different levels of neurocognitive functioning in Alzheimer’s disease course

**DOI:** 10.1186/s13195-020-00624-3

**Published:** 2020-05-08

**Authors:** Claudio Liguori, Fabio Placidi, Francesca Izzi, Matteo Spanetta, Nicola Biagio Mercuri, Alessandra Di Pucchio

**Affiliations:** 1 Sleep Medicine Centre, Department of Systems Medicine, University of Rome ‘Tor Vergata”, Rome, Italy; 2 Neurology Unit, Department of Systems Medicine, University of Rome ‘Tor Vergata”, Viale Oxford, 81, 00133 Rome, Italy; 3 Fondazione Santa Lucia IRCCS, Rome, Italy; 4 Training Office, Italian National Institute of Health (Istituto Superiore di Sanità), Rome, Italy

**Correction to: Alz Res Therapy 12, 5 (2020)**

**https://doi.org/10.1186/s13195-019-0571-3**

Following publication of the original article [1], the authors reported two errors in the Results section.

1. The sentence “TST was lower in CN, SCI, and MCI compared to mAD patients who in turn showed a lower TST than msAD patients.” under subsection “PSG data (one-way ANOVA analysis)”should be replaced with:

TST was HIGHER in CN, SCI, and MCI compared to mAD patients who in turn showed a HIGHER TST than msAD patients.

2. The sentence “Was excluded as it presented split loadings on all factors, and SE and TIB were excluded as they were computed from other polysomnographic parameters.” under subsection “Principal component analysis” should be removed.
